# Study on the structure of pitch-polymer compositions by fluorescence microscope

**DOI:** 10.1007/s00396-014-3413-7

**Published:** 2014-10-16

**Authors:** Grzegorz Makomaski

**Affiliations:** Institute of Chemistry in Plock, Warsaw University of Technology, Lukasiewicza 17, 09-400 Plock, Poland

**Keywords:** Waste polymers, Pitch-polymer compositions, Fluorescence microscope, Colloidal structure

## Abstract

In this work, the results of studies on the evaluation of colloidal structure of coal-tar pitch compositions with selected waste polymers by fluorescence microscope. For pitch-polymer compositions containing 10–50 wt% waste polymer, softening point, coking value and content of components insoluble in toluene and quinoline were carried out. The results indicate that pitch-polymer compositions can be treated as microheterogeneous systems, colloidal and biphase, generally exhibiting uniform dispersion of particles composed of polymer macromolecules and probably of α components of coal-tar pitch.

## Introduction

Coal-tar pitch is an important feedstock of carbochemical origin used for the production of i.a. carbon anodes, graphite electrodes, fireproof materials, carbon-carbon composites and carbon fibres, as coking additive and as binder in many insulating and sealing materials used in construction and road building [1, 2]. Bitumens originating from coal, similarly as petroleum asphalts can be treated as colloidal-dispersive systems. Micelles composed of α_2_ components and partly of α_1_ components form dispersed phase while oils (γ components) and partly β components are the dispersing phase [3].

Because of the presence of carcinogenic aromatic hydrocarbons in coal-tar pitch, there is a need for its modification to obtain material safe for natural environment. Research on the preparation of modified bituminous substances originating from coal, carried out for many years in the Institute of Chemistry of Warsaw University of Technology in Plock has led to the elaboration of method reducing the level of carcinogenicity of coal-tar pitch [4]. Modification of coal-tar pitch with polymers influences also significantly its group composition and properties. The direction and scale of changes depend on chemical structure of the modifier and its amount. Chemical structure is a factor directly determining properties of each of the components of bitumen-polymer compositions and simultaneously has an impact on the possibility to form a specific structure of a given composition, on which in turn its properties depend [5–9].

Bitumen-polymer mixtures find wider and wider use in modern technology. Because of profitable properties of pitch-polymer mixtures, it would be advisable to check the possibilities to use waste polymers for the modification of the pitch. It leads to the search for new techniques and methods of evaluation of their homogenicity and application properties. One of them are microscopic studies, which play significant role in the determination of the structure and colloidal stability of bitumens and bitumen-polymer systems [10], including pitch-polymer compositions [7]. In the studies of bitumen-polymer systems, fluorescence microscope is used, particularly for the evaluation of polymer-asphalt materials [11–13]. However, there is no significant development in the evaluation of pitch-polymer compositions with the use of such microscope.

The aim of this work was to determine the utility of fluorescence microscope for the evaluation of the structure of coal-tar pitch compositions with selected waste polymers and to study their influence on pitch properties.

## Experimental

The raw materials used in this study were coal-tar pitch (CTP) and selected waste polymers: poly(ethylene terephthalate) (PET), poly(methylene methacrylate) (PMMA) and phenol-formaldehyde resin (PF). Pitch-polymer compositions containing from 10 to 50 wt% waste polymer were prepared in the conditions allowing to obtain homogeneous and stable mixtures. Depending on the applied waste polymer, the components were homogenized in the temperatures from 150 to 270 °C, during 0.5–2.5 h. The homogenization of the composition components was carried out at as low as possible temperatures (from which it was possible to mix the components). Idea was to eliminate of the destruction and/or degradation processes of waste polymers in coal-tar pitch during the preparation of compositions. The composition of mixtures and preparation conditions are presented in Table 1.

**Table 1:** Preparation conditions and properties of pitch-polymer compositions

Compositions (wt%)	Preparation of compositions	SP (°C)	CV (wt%)	TI (wt%)	QI (wt%)
CTP	−	107.0	53.03	34.21	7.14
90 CTP + 10 PET	260 °C 0.5 h	127.0	53.62	45.80	13.31
75 CTP + 25 PET		166.0	50.10	43.10	29.43
50 CTP + 50 PET		236.0	38.00	68.50	62.79
90 CTP + 10 PMMA	270 °C 1 h	137.0	60.08	35.62	8.78
75 CTP + 25 PMMA		145.0	48.33	29.36	9.22
50 CTP + 50 PMMA		200.0	28.79	23.03	10.49
90 CTP + 10 PF	150 °C 2.5 h	128.0	54.16	55.18	10.14
75 CTP + 25 PF		141.0	53.67	63.04	4.48
50 CTP + 50 PF		*	54.07	88.54	4.21

*Infusible composition

For coal-tar pitch and pitch-polymer compositions, the following measurements were carried out:

- softening point by “Ring and Ball” method (SP) according to the PN-EN 1427:2009 standard,
- coking value (CV) according to the PN-C-97093:1993 standard,
- content of components insoluble in quinoline (QI) according to the PN-C-97058:1999 standard and
- content of components insoluble in toluene (TI) according to the method elaborated in the Institute of Chemistry, Warsaw University of Technology in Plock.

The colloidal structure of pitch-polymer compositions by Olympus BX41 microscope was carried out. In the study of colloidal structure of pitch-polymer compositions, in ultraviolet light, press moulding of compositions on the microscopic slide by a hydraulic press was on adopted technique of preparing of samples for study. Parameters of press moulding of samples: pressure from 2.5 to 3.5 MPa, time of press moulding 5 min, temperature of press moulding from 130 to 190 °C.

## Results and discussion

### Pitch-poly(ethylene terephthalate) compositions

Selected results of measurements of physicochemical properties of coal-tar pitch and pitch-poly(ethylene terephthalate) compositions are presented in Table 1.

The addition of waste PET significantly influenced the softening point of CTP. With the increase of PET in the compositions, the softening point increased. For the composition containing 50 wt% of PET, the softening point compared to the unmodified pitch increased by 129 °C.

With the increase of waste PET in the compositions, the cooking value decreased and so the yield of residue after high-temperature carbonization process. In particular, significant changes occurred for compositions containing 50 wt% of the waste, where coking value was lower by 15 wt% compared to coal-tar pitch.

Addition of waste PET into CTP cased changes of group composition of the bitumen. The content of TI component in pitch-PET compositions was 45–69 wt% and QI components was 13–63 wt%. With the increase of waste in the compositions, the content of TI and QI components increased.

**Fig. 1:**
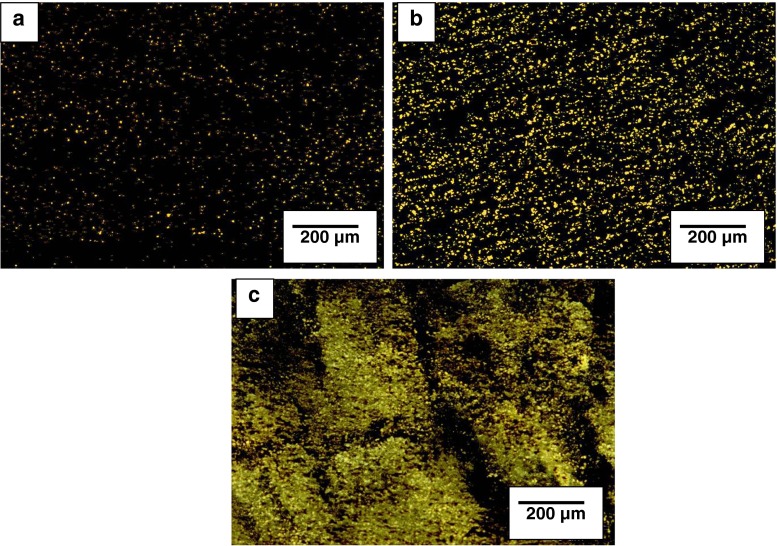
Microscopic images of the structure of pitch-PET compositions containing the following: **a** 10 wt% of waste PET, **b** 25 wt% of waste PET, and **c** 50 wt% of waste PET

In Fig. 1, microscopic images of pitch-PET compositions obtained using fluorescence microscope are presented. On obtained microscopic images, similarly as in the case of petroleum bitumens, carbon bitumen phase is observed in black colour.

Structures of compositions containing ≤25 wt% PET, had uniform level of dispersion, and dispersed polymer particles had round and regular shapes. The increase of waste PET amount in compositions caused the increase of the amount and size of dispersed particles. For the composition containing 50 wt% of waste poly(ethylene terephthalate) agglomerates of irregular shape could be observed, which were formed from aggregates containing polymer macromolecules and probably α components of coal-tar pitch. Phase inversion of the dispersion was observed. It has to be presumed that with the increase of waste PET amount in compositions, the development of micellar areas occurred due to strong physical interactions between the components, which is indicated by i.a., high softening temperatures.

### Pitch-poly(methylene methacrylate) compositions

Selected results of measurements of physicochemical properties of pitch-poly(methylene methacrylate) compositions are presented in Table 1.

Addition of waste PMMA influenced the change of physicochemical properties of coal-tar pitch. The increase of waste PMMA addition into CTP caused the increase of softening point. For the compositions containing 10 or 25 wt% of waste PMMA, the softening point compared to the unmodified pitch increased by 30 and 38 °C, respectively. The highest softening point was observed for the compositions containing 50 wt% of waste PMMA.

Addition of waste PMMA into CTP caused the decrease of coking value and so the yield of residue after high-temperature carbonization process. An exemption from this rule was the composition containing 10 wt% of waste PMMA, which had higher coking value than CTP (by 7.05 wt%). In particular, significant changes occurred for compositions containing 50 wt% of the waste, where coking value was lower by 24.20 wt% compared to coal-tar pitch.

Increase of waste PMMA addition into CTP caused the decrease of content of TI components and increase of content of QI components. The pitch-PMMA compositions had lower content of TI components and had higher content of QI components compared to coal-tar pitch. An exemption from this rule was the composition containing 10 wt% of waste PMMA, which had higher content of TI components by 1.41 wt% compared to CTP.

**Fig. 2:**
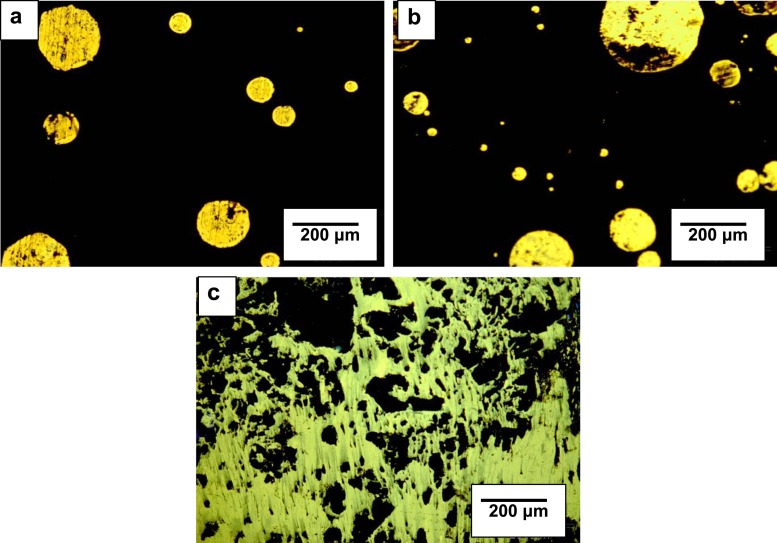
Microscopic images of the structure of pitch-PMMA compositions containing the following: **a** 10 wt% of waste PMMA, **b** 25 wt% of waste PMMA, **c** 50 wt% of waste PMMA

In Fig. 2, microscopic images of pitch-PMMA compositions obtained using fluorescence microscope are presented.

Structures of pitch-waste PMMA compositions containing up to 25 wt% of additive assumed the shapes of round particles of different shapes, chaotically dispersed in continuous phase of the bitumen. With the increase of waste PMMA in compositions increased the amount and size of dispersed aggregates of irregular shapes, composed of macromolecules of a polymer and probably of α components of coal-tar pitch. For the composition containing 50 wt% of waste PMMA, phase inversion was observed in the dispersion.

### Pitch-phenol-formaldehyde resin compositions

Selected results of measurements of physicochemical properties of pitch-phenol-formaldehyde resin compositions are presented in Table 1.

Compositions containing waste PF had increased softening points, compared to CTP. Softening point increased with increasing content of waste PF. In the case of compositions containing 50 wt% of this component, the measurements of softening point was not possible because of the impossibility to melt them. For the compositions containing PF, independently from the amount of waste, coking values were similar to the value of coal-tar pitch.

Addition of waste PF into CTP caused changes of group composition of the bitumen. With the increase of waste PF in the compositions, the content of TI components increased. For the composition containing 50 wt% of waste PF, the content of TI components increased by 54.33 wt% compared to the unmodified pitch. The content of QI components in pitch-PF compositions was 4.2–4.5 wt% and was lower than CTP. An exemption from this rule was the composition containing 10 wt% of waste PF, which had higher content of QI components compared to coal-tar pitch (by 3 wt%).

**Fig. 3:**
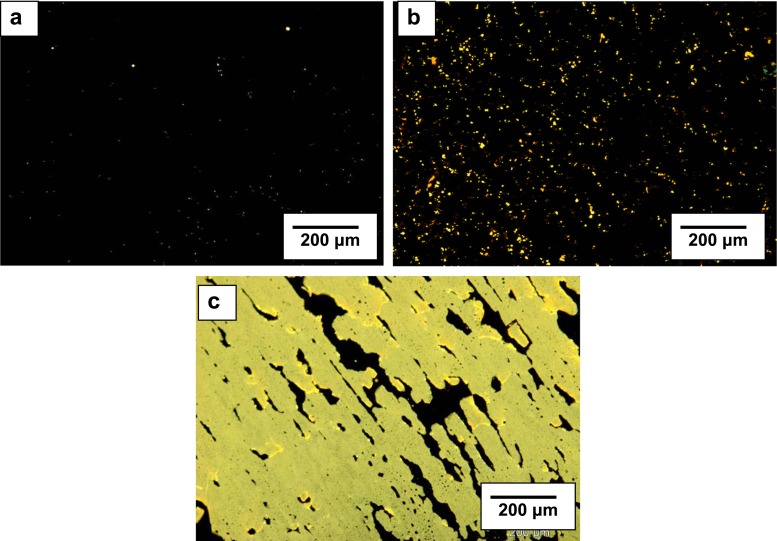
Microscopic images of the structure of pitch-PF compositions containing the following: **a** 10 wt% of waste PF, **b** 25 wt% of waste PF, **c** 50 wt% of waste PF

In Fig. 3, microscopic images of pitch-PF compositions obtained using fluorescence microscope are presented. Structures of compositions containing ≤25 wt% of PF, similarly as in the case of compositions containing PET in the amount of ≤25 wt%, exhibited uniform level of dispersion, and dispersed particles had round shape. Further increase of the amount of phenol-formaldehyde resin in compositions up to 50 wt% caused formation of agglomerates of irregular shapes, being assemblies of aggregates composed of polymer macromolecules and probably of α components of coal-tar pitch (Fig. 3).

## Conclusions

The application of fluorescence microscope for the evaluation of colloidal structure of pitch-polymer compositions can be a valuable supplement to other methods used to evaluate the homogenicity and level of dispersion of a polymer in bitumen-polymer compositions, and also it can allow to explain the phenomena occurring during processes of modification of coal-originated bitumens with macromolecular compounds.

The changes of physicochemical properties of coal-tar pitch depended on the type and amount of applied waste polymer. Addition of waste PET into CTP caused increase of softening point and the amount of components insoluble in toluene and quinoline, while coking value in pitch-PET compositions decreased. Addition of waste PMMA into CTP caused increase softening point and the amount of components insoluble in quinoline, decrease coking value and the amount of components insoluble in toluene. Addition of waste PF into CTP caused increase softening point and the amount of components insoluble in toluene, decrease the amount of components insoluble in quinoline and caused slightly changes of coking value.

Pitch-polymer compositions can be treated as microheterogeneous systems, colloidal and biphase, generally exhibiting uniform dispersion of particles composed of polymer macromolecules and probably of α components of coal-tar pitch. The size of dispersed particles depends on the type and amount of waste polymer added to the coal-tar pitch.

Observed microscopic images allowed to point out the following dependences between the properties of pitch-polymer compositions and their structure:

- for pitch-PET compositions, the increase of the size of dispersed aggregates caused the increase of softening point and content of TI and QI components and reduction of coking value,
- for compositions containing waste PMMA, the increase of the size of dispersed aggregates caused the increase of softening point and content of QI components, reduction of coking value and content of TI components and
- for pitch-PF compositions, the increase of the size of dispersed aggregates caused the increase of softening point and content of TI components and decrease content of QI components, while coking value changed insignificantly.
